# Supplementary material for: Nitro-Oleic Acid Inhibits Stemness Maintenance and Enhances Neural Differentiation of Mouse Embryonic Stem Cells via STAT3 Signaling
Source: Int J Mol Sci. 2021 Sep 15;22(18):9981. doi: 10.3390/ijms22189981 (PMC8468660; doi:10.3390/ijms22189981)
Supplement: Supplementary file 1 [file ijms-22-09981-s001.zip › Pereckova-2021-supplementary data.pdf]

International Journal of *Molecular Sciences*

MDPI

Supplemented data

Nitro-oleic acid inhibits stemness maintenance and enhances neural differentiation of mouse embryonic stem cells via STAT3 signaling

Jana Pereckova[1]*, Michaela Pekarova[1], Nikoletta Szamecova[1,2], Zuzana Hoferova[1], Kristyna Kamarytova[1,2], Martin Falk[1], and Tomas Perecko[1]

[1] Institute of Biophysics of the Czech Academy of Sciences, Department of Cell Biology and Radiobiology, Kralovopolska 135, 612 65 Brno, Czech Republic

[2] Department of Biochemistry, Faculty of Science, Masaryk University, Kamenice 5, 625 00 Brno, Czech Republic

* Correspondence: Jana Pereckova, Institute of Biophysics, Academy of Sciences of the Czech Republic, Kralovopolska 135, 612 65 Brno, Czech Republic. Email address: pereckova@ibp.cz, Fax: +420 - 541 211 101

ORCID:
Jana Pereckova: 0000-0002-7565-993X
Michaela Pekarova: 0000-0003-1252-455X
Martin Falk: 0000-0002-9229-0468
Tomas Perecko: 0000-0003-4278-4088

Citation: Pereckova, J.; Pekarova, M.; Szamecova, N.; Hoferova, Z.; Kamarytova, K.; Falk, M.; Perecko, T. Nitro-Oleic Acid Inhibits Stemness Maintenance and Enhances Neural Differentiation of Mouse Embryonic Stem Cells via STAT3 Signaling. *Int. J. Mol. Sci.* **2021**, *22*, 9981. https://doi.org/10.3390/ijms22189981
Academic Editor: Irmgard Tegeder

Received: 16 April 2021
Accepted: 12 September 2021
Published: 15 September 2021

Publisher's Note: MDPI stays neutral with regard to jurisdictional claims in published maps and institutional affiliations.

Copyright: © 2021 by the authors. Submitted for possible open access publication under the terms and conditions of the Creative Commons Attribution (CC BY) license (http://creativecommons.org/licenses/by/4.0/).

1. Methods

1 .1. Cytotoxicity assay

To determine the cytotoxicity of nitro oleic acid (NO_2-OA) on mouse embryonic stem cells (mESC), the cells were cultured in complete medium with or without leukemia inhibitory factor on gelatin-coated 24-well plates. Duplicate wells were exposed to NO_2-OA at concentration of 0, 1, and 10 μM. After 72 h, 3-(4,5-dimethylthiazol-2-yl)-2,5-diphenyltetrazolium bromide (MTT, Sigma) assay was performed. MTT was added to cells at 2.5 mg/ml. After 2 h, the medium was aspired and 10 % Triton X-100 was added. The quantity of formazan was measured after 5 min incubation by the absorbance at 570 nm using a plate reading spectrophotometer.

2. Figures

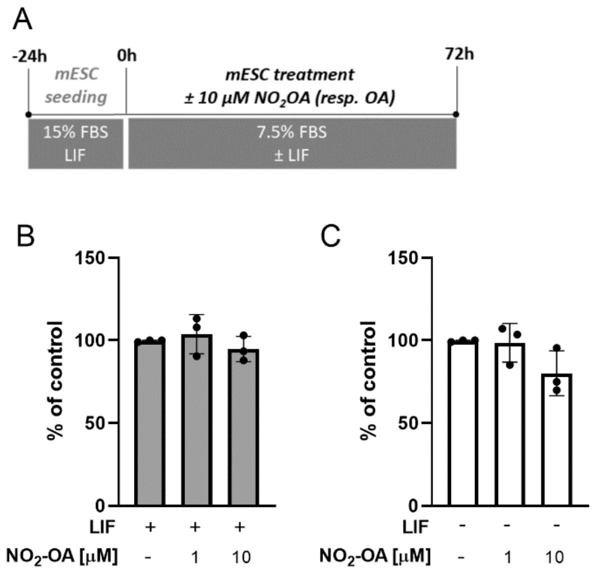

Figure S1: Cytotoxic effects of NO_2-OA treatment on mouse embryonic stem cells (mESC). Cytotoxicity of NO_2-OA was analyzed after 72h of incubation at different concentration (0, 1, and 10 μM), with (B) or without (C) leukemia inhibitory factor (LIF). Data passed Shapiro-Wilk test for normal distribution. Results are expressed as means and standard deviation from three independent experiments. Statistical significance was determined by Unpaired Student's t-test; statistical differences compare to non-treated control were analyzed.

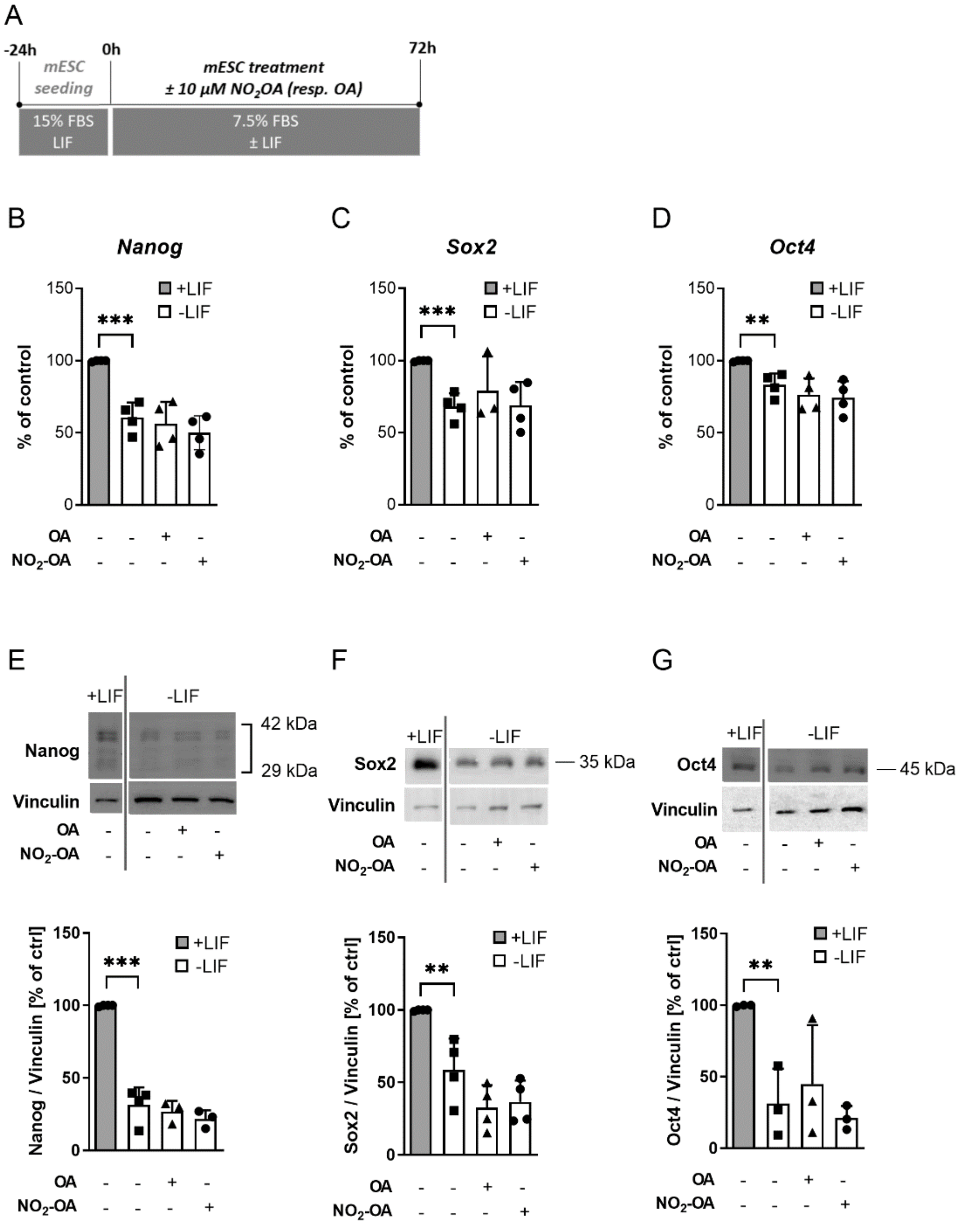

Figure S2: NO2-OA does not affect the expression of pluripotency markers without the presence of leukemia inhibitory factor (LIF) in mouse embryonic stem cells (mESC). (A) Time-scheme of the mESC treatment used in this experiment. (B-G) The levels of selected markers of pluripotency; (B) Nanog, (C) Sex-determining region Y-box 1 transcription factor (Sox2), and (D) octamer-binding transcription factor 4 (Oct4) were evaluated by real-time quantitative PCR (B-D) and western blot (E-G) in mESC incubated for 72 h in experimental medium ± leukemia inhibitory factor + 7.5% FBS and treated by NO2-OA or OA (10 μM). Data passed Shapiro-Wilk test for normal distribution. Results are expressed as means and standard deviation from at least three independent experiments. Statistical significance was determined by Unpaired Student's t-test; statistical differences compare to corresponding non-treated control were analyzed, $^{**}p<0.01$, $^{***}p<0.001$.

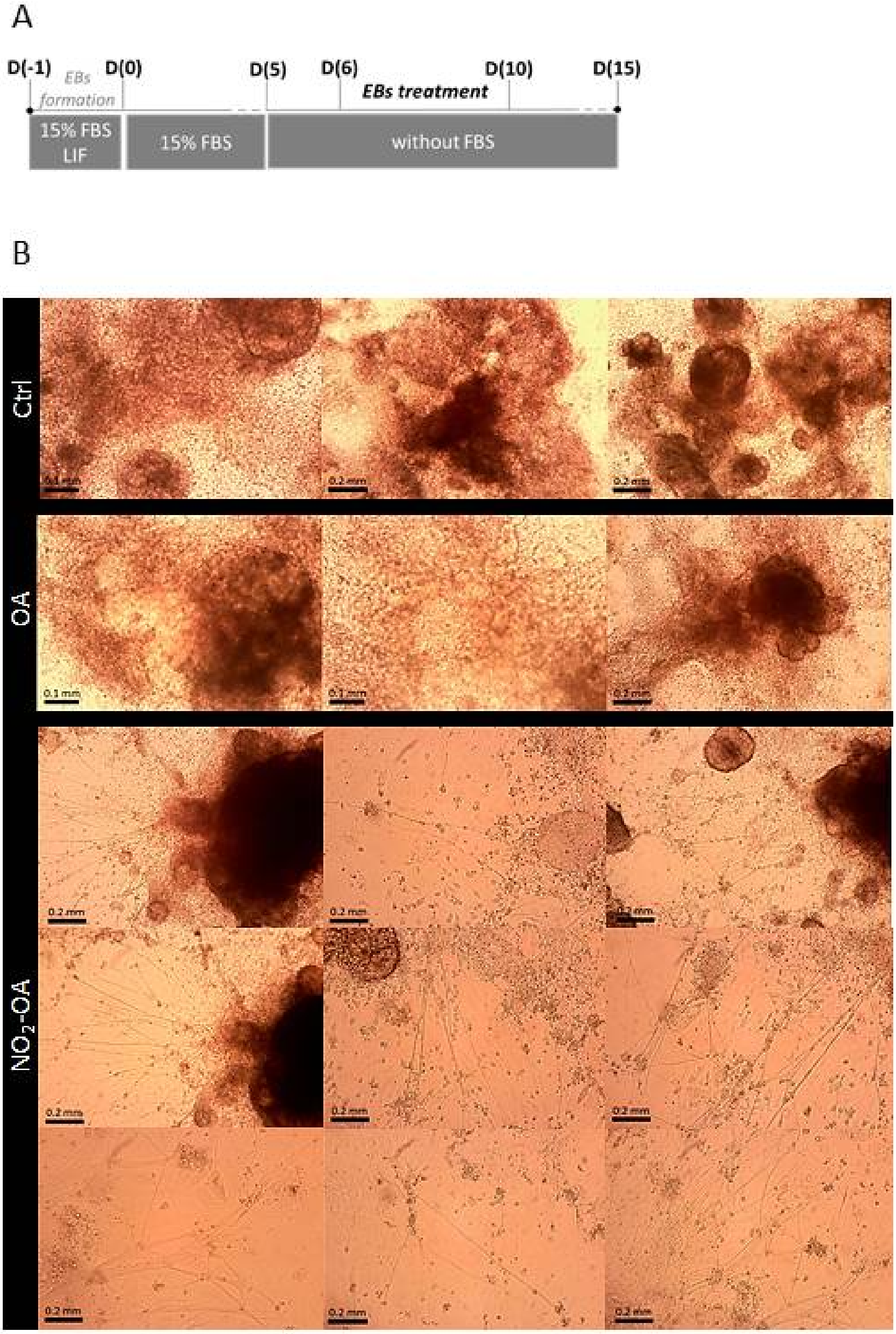

Figure S5: NO_2-OA enhances neural differentiation in mESC-derived EBs. The morphological study was performed using light microscope. (A) Time-scheme of the mESC treatment used in this experiment. (B) Cells in control (Ctrl), OA and NO_2-OA groups were analyzed at day 14 of EBs differentiation.
